# Longitudinal associations between amyloid and symptoms of depression and anxiety in subjective cognitive decline: the impact of personality characteristics

**DOI:** 10.3389/fpsyt.2025.1572174

**Published:** 2025-06-10

**Authors:** Calvin Trieu, Mardou S. S. A. van Leeuwenstijn, Lisa-Marie Schlüter, Jarith L. Ebenau, Inge M. W. Verberk, Sietske A. M. Sikkes, Sander C. J. Verfaillie, Elsmarieke van de Giessen, Charlotte E. Teunissen, Wiesje M. van der Flier, Argonde C. van Harten

**Affiliations:** ^1^ Alzheimer Center Amsterdam, Department of Neurology, Amsterdam University Medical Center (UMC) location Vrije Universiteit Medical Center (VUmc), Vrije Universiteit Amsterdam, Amsterdam, Netherlands; ^2^ Amsterdam Neuroscience, Neurodegeneration, Amsterdam, Netherlands; ^3^ Neurochemistry Laboratory, Department of Clinical Chemistry, Amsterdam Neuroscience, Program Neurodegeneration, Amsterdam University Medical Center (UMC), Vrije Universiteit Amsterdam, Amsterdam, Netherlands; ^4^ Faculty of Behavioural and Movement Sciences, Clinical Developmental Psychology & Clinical Neuropsychology, Vrije Universiteit Amsterdam, Amsterdam, Netherlands; ^5^ Department of Radiology & Nuclear Medicine, Amsterdam Neuroscience, Vrije Universiteit Amsterdam, Amsterdam University Medical Center (UMC), Amsterdam, Netherlands; ^6^ Department of Medical Psychology, Amsterdam Public Health Research Institute, University of Amsterdam, Amsterdam University Medical Center (UMC), Amsterdam, Netherlands; ^7^ Department of Psychiatry, Amsterdam University Medical Center (UMC) location Universiteit Medical Center (VUmc), Amsterdam, Netherlands

**Keywords:** Alzheimer’s disease, depression, anxiety, subjective cognitive decline, neuroticism, somatization

## Abstract

**Introduction:**

Depressive/anxiety symptoms are common in subjective cognitive decline (SCD) and may relate to Alzheimer’s pathology, potentially modulated by personality characteristics.

**Methods:**

Depressive/anxiety symptoms were assessed over 4 ± 2 years in 329 SCD (88 amyloid-positive/241 amyloid-negative) using Geriatric Depression Scale-15 (GDS), Center for Epidemiological Studies-Depression (CES-D), and Hospital Anxiety and Depression Scale-Anxiety (HADS-A). Mixed-effects models assessed associations between amyloid status and these symptoms, with neuroticism and somatization as effect-modifiers.

**Results:**

Amyloid status was not directly associated with GDS, CES-D or HADS-A. However, neuroticism modified the association between amyloid status and GDS (p<0.05). In lower neuroticism, amyloid positivity was associated with GDS increase (β:0.10 ± 0.08), but not in higher neuroticism (β:-0.04 ± 0.12). Somatization modified the association between amyloid status and CES-D (p<0.05). In lower somatization, amyloid positivity was associated with CES-D increase (β:0.65 ± 0.23), but not in higher somatization (β:-0.12 ± 0.29).

**Discussion:**

Amyloid-positive individuals with lower neuroticism/somatization increased more in depressive symptoms over time, suggesting a preclinical AD-related depressive phenotype.

## 1 Introduction

Subjective cognitive decline (SCD) has gained increasing interest as a potential early stage of Alzheimer’s Disease (AD). SCD is defined by self-reported cognitive decline, in the absence of objective cognitive impairment (1). SCD can occur as one of the earliest symptomatic expressions of underlying AD (also recognized as stage 2 AD). More often however, SCD is not related to early neurodegenerative diseases, but rather to personality features, psychiatric disorders, lifestyle factors, or systemic illnesses (2–5).

In addition to the subjective experience of cognitive decline, individuals with SCD frequently exhibit symptoms of depression and anxiety (9–11), which can already compromise daily functioning even at the earliest stages of cognitive decline (12, 13). Previous studies report an association between amyloid pathology and symptoms of depression and anxiety in cognitively unimpaired individuals (14–16). However, findings in individuals with SCD were more inconsistent, with some studies identifying an association between amyloid pathology and symptoms of depression and anxiety, while other studies did not (17–20). The origin of symptoms of depression and anxiety in individuals with SCD is multifaceted and may be influenced by various factors (21). These symptoms may be underpinned by pathophysiological alterations associated with AD, such as the accumulation of beta-amyloid and tau, and could therefore be a direct symptom of AD (22). Alternatively, symptoms of depression and anxiety may be entirely unrelated to underlying AD pathology. Personality characteristics such as neuroticism which involves a tendency towards negative emotions, and somatization, characterized by experiencing psychological distress as somatic symptoms, may influence and exacerbate how individuals with SCD experience symptoms of depression and anxiety (6). Additionally, previous evidence suggests that neuroticism may increase susceptibility to Alzheimer’s disease pathology and its progression, although the nature of this association has not been fully elucidated (7, 8). Given prior evidence that individuals with SCD have higher levels of neuroticism and poorer perceived physical health, we hypothesized that neuroticism and somatization may influence the trajectories of symptoms of depression and anxiety in this population and potentially serve as effect modifiers in the association between amyloid status and symptoms of depression and anxiety (23).

Our aims were to investigate (1) whether amyloid status at baseline is associated with the longitudinal course of symptoms of depression and anxiety in individuals with SCD and (2) the influence of neuroticism and somatization on symptoms of depression and anxiety and their modulating effect on the association between amyloid status and symptoms of depression and anxiety.

## 2 Methods

### 2.1 Participants

We included 329 participants with SCD from the Subjective Cognitive Impairment Cohort (SCIENCe), embedded within the Amsterdam Dementia Cohort at the Alzheimer Center Amsterdam, Amsterdam UMC for whom baseline amyloid status had been assessed using cerebrospinal fluid (CSF) or positron emission tomography (PET). SCIENCe participants were recruited from our tertiary memory clinic, where they were primarily referred by their general physician, neurologist or geriatrician. The main inclusion criteria of SCIENCe are (1) a diagnosis of SCD, defined as the subjective experience of cognitive decline, without objective cognitive impairment on neuropsychological testing across all cognitive domains, including memory, language, attention/processing speed, and executive functioning, and (2) an age of 45 years or older. Exclusion criteria were (1) cognitive complaints caused by mild cognitive impairment, dementia or other neurological diseases or major psychiatric disorders as defined by the DSM-5, including individuals with a GDS score above 6 and evidence of a depressive disorder (2) history of alcohol/substance abuse. A full description of the SCIENCe inclusion and exclusion criteria has been published previously (24). All included participants underwent a standardized workup at baseline that included an assessment of their medical history, physical examination, neurological examination, psychological questionnaires, neuropsychiatric questionnaires, neuropsychological assessments, laboratory testing, and cerebrospinal fluid (CSF) and/or amyloid positron emission tomography (PET). During annual follow-up visits, we repeated the neuropsychiatric questionnaires and neuropsychological assessments, and reevaluated diagnoses to determine whether participants had progressed to MCI, AD dementia or other types of dementia (i.e., vascular dementia, dementia with Lewy bodies or frontotemporal dementia). On average, the follow-up time was 4±2 years.

### 2.2 Amyloid status

We determined amyloid status of all participants using amyloid PET (n=231) or CSF biomarkers (n=98). When both PET and CSF were available, we used PET to determine amyloid status. Amyloid PET scans were conducted using either [^18^F]florabetapir (n=144), [^18^F]florabetaben (n=80), [^18^F]flutemetamol (n=5) or [^11^C]Pittsburgh compound-B (n=2) radiotracers, and were acquired on the following systems: Gemini TF PET/CT, Ingenuity TF PET/CT, and Ingenuity PET/MRI (Philips Healtcare, Best, The Netherlands). A more detailed description of the PET protocol is provided elsewhere (24, 25). Trained nuclear medicine physicians visually rated the amyloid PET scans as “amyloid positive” or “amyloid negative”. CSF was obtained by performing a lumbar puncture at the L3/L4, L4/L5 or L5/S1 intervertebral space, using an atraumatic 25-gauge needle, which was then collected into a polypropylene tube, centrifuged, aliquoted into 0.5ml and stored at -80°C until further analysis. CSF was analyzed using either Innotest ELISA (n=58, Innogenetics-Fujirebio, Ghent, Belgium) or Elecsys (n=40, Roche Diagnostics GmbH, Mannheim, Germany). Innotest Aβ_42_ values were adjusted in the CSF biomarker analyses, to account for drift that occurred over the years (26). Amyloid positivity was defined as a drift-corrected concentration of <813 pg/ml for Innotest (26) and as a pTau/Aβ_42_ ratio of >0.02 for Elecsys (27).

### 2.3 Symptoms of depression and anxiety

We assessed symptoms of depression using the Geriatric Depression Scale-15 (GDS) and the Center for Epidemiological Studies Depression scale (CES-D), both self-reported questionnaires (28, 29). The GDS consists of 15 items with a total score ranging from 0 to 15, and the CES-D consists of 20 items with a score ranging from 0 to 60, with higher score indicating more severe symptoms of depression for both questionnaires. We assessed symptoms of anxiety using the Hospital Anxiety & Depression Scale – Anxiety subscale (HADS-A) (30). The HADS-A is a self-reported screening tool, which consists of 7 items, rating on a scale from 0 to 3. The total score of the HADS-A ranges from 0 to 21, with higher score indicating more severe symptoms of anxiety. During the study duration, we obtained a total of 1015 GDS datapoints, with a median of 3 measurements per participant. For CES-D and HADS-A, we obtained 1484 and 1485 datapoints, respectively, with a median of 4 measurements per participant.

### 2.4 Personality characteristics

At baseline, we assessed neuroticism using the Dutch Personality Questionnaire – Neuroticism subscale (DPQ-N) (31). The DPQ-N was shortened from 20 to 15 items, with a total score ranging from 0 to 30, with higher scores indicating a greater level of neuroticism. We assessed somatization using the Four-Dimensional Symptom Questionnaire – Somatization subscale (4DKL-S) (32). The 4DKL-S consists of 16 items, with total score ranging from 0 to 32, with higher score indicating a higher level of somatization.

### 2.5 Statistical analysis

All statistical analyses were performed in Rstudio version 2022.12.0 (RStudio, Inc., Boston, MA, USA), with packages ‘lme4’ version 1.1–30 and ‘lmtest’ version 0.9-40. To compare differences in demographic characteristics, we used the independent t-test, Mann-Whitney U test or Pearson χ2, depending on the data distribution. First, we used linear mixed models (LMM) with only time as a predictor to examine the trajectory of symptoms of depression and anxiety over time in the total group. Next, we analyzed associations between amyloid status and symptoms of depression and anxiety with models including terms for amyloid status (positive/negative), time and amyloid status*time interaction. Separate models were used with GDS, CES-D and HADS-A as outcome variable. Similarly, we used LMMs to analyze associations between neuroticism and somatization (separate models) and symptoms of depression and anxiety, with terms for each personality characteristic, time and personality characteristic*time. Subsequently, we investigated the potentially modifying effect of neuroticism and somatization, constructing models including terms for amyloid status, time, effect modifier (neuroticism or somatization), and amyloid status*time*effect modifier interaction. When the three-way interaction term (amyloid status*time*effect modifier) was significant (p<0.05), we considered this as evidence of effect modification and used a median split of the effect modifier to visualize the interaction and to perform *post hoc* analyses stratified by “low” and “high” levels of neuroticism and somatization to explore the nature of the interaction effect.

## 3 Results

### 3.1 Demographic characteristics

We included 329 participants, of whom 88 (26.7%) were amyloid positive (A+) and 241 (73.3%) were amyloid negative (A-; **Table 1**). A+ participants (66.2±7.0) were somewhat older than A- participants (61.4±7.5; p<0.001). Somatization (4DKL-S) was lower in the A+ group (4.9±4.2) than in the A- group (6.4±5.2; p<0.05). Distribution of sex, educational level, baseline MMSE, and neuroticism (DPQ-N) did not differ according to amyloid status. At baseline, symptoms of depression and anxiety across the entire group were: GDS 2.6±2.1, CES-D 9.0±6.6, and HADS-A 4.0±2.9. Longitudinally, CES-D slightly increased over time (β±SE_Time_ 0.18±0.07; p<0.05), while the scores of GDS and HADS-A remained stable over time (β_Time_ -0.01±0.03, -0.02±0.03). The demographic and clinical characteristics of the included participants are summarized in **Table 1**.

**Table 1:** Demographic characteristics.

Characteristic	Total (n = 329)	A+ (n = 88)	A- (n = 241)	P-values
Age, mean (SD)	62.7 (7.7)	66.2 (7.0)	61.4 (7.5)	<0.001
Female, n (%)	137 (41.6)	36 (40.9)	101 (41.9)	N.S.
Education, median (IQR)	6.0 (5.0-6.0)	6.0 (5.0-7.0)	6.0 (5.0-6.0)	N.S.
APOE ϵ4 genotype, n (%)	134 (40.7)	58 (65.9)	76 (31.5)	<0.001
MMSE, mean (SD)	28.6 (1.3)	28.5 (1.2)	28.6 (1.4)	N.S.
MoCA, mean (SD)	26.2 (2.4)	26.3 (2.6)	26.1 (2.4)	N.S.
GDS, mean (SD)	2.6 (2.1)	2.3 (2.1)	2.7 (2.1)	N.S.
CES-D, mean (SD)	9.0 (6.6)	8.4 (6.1)	9.2 (6.8)	N.S.
HADS-A, mean (SD)	4.0 (2.9)	4.2 (3.1)	3.9 (2.8)	N.S.
DPQ-N, mean (SD)	6.2 (5.3)	5.7 (5.3)	6.4 (5.3)	N.S.
4DKL-S, mean (SD)	6.0 (5.0)	4.9 (4.2)	6.4 (5.2)	<0.05
Family history of dementia, n (%)	178 (54.1)	57 (64.8)	121 (50.2)	<0.05
Family history of psychiatry, n (%)	83 (25.2)	19 (21.6)	64 (26.6)	N.S.

Demographic differences between groups were calculated with the independent t-test, Mann-Whitney U test or Pearson χ2 test as appropriate.

APOE ϵ4, Apolipoprotein E ϵ4; MMSE, Mini-Mental State Examination; MoCA, Montreal Cognitive Assessment; GDS, Geriatric Depression Scale; CES-D, Center for Epidemiologic Studies Depression Scale; HADS-A, Hospital Anxiety and Depression Scale - Anxiety; DPQ-N, Dutch Personality Questionnaire - Neuroticism; 4DKL-S, Four-Dimensional Symptom Questionnaire – Somatization, N.S., not significant.

### 3.2 Associations between amyloid status and symptoms of depression and anxiety

**Figure 1:**
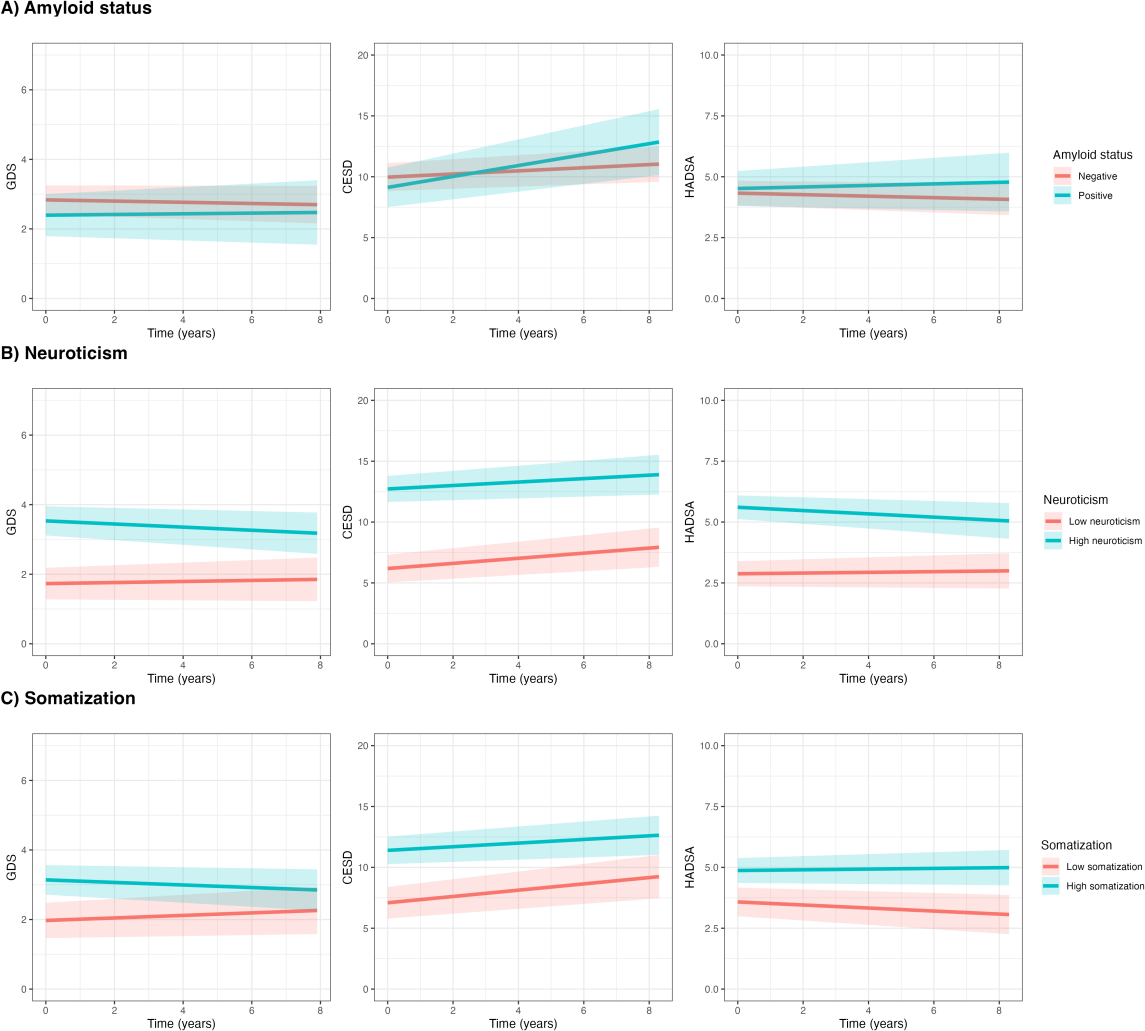
Associations between amyloid status & personality characteristics and symptoms of depression and anxiety over time. Figures show symptom trajectories of depression and anxiety over 8 years based on linear mixed models for GDS (left column), CES-D (middle column), and HADS-A (right column), with shaded areas representing 95% confidence intervals. **(A–C)** show comparisons by amyloid status (red = negative, blue = positive), neuroticism, and somatization (red = low, blue = high), with neuroticism and somatization groups defined by a median split. GDS, Geriatric Depression Scale; CES-D, Center for Epidemiologic Studies Depression Scale; HADS-A, Hospital Anxiety and Depression Scale – Anxiety.

Univariate LMMs showed no significant associations between amyloid status and GDS, CES-D and HADS-A at baseline or over time (**Table 2**, **Figure 1A**). However, we observed a trend towards a more pronounced increase in CES-D over time in A+ than in A- (β_Time*Amyloid status_ 0.32 ± 0.19; p=0.088).

**Table 2:** Linear mixed models of the association between amyloid status & personality characteristics and symptoms of depression and anxiety.

Terms	GDS		CES-D		HADS-A	
	Baseline	Change over time	Baseline	Change over time	Baseline	Change over time
Amyloid status	-0.44 ± 0.32	0.03 ± 0.07	-0.84 ± 0.86	0.32 ± 0.19^	0.19 ± 0.38	0.06 ± 0.08
Neuroticism	0.21 ± 0.02**	-0.01 ± 0.01*	0.75 ± 0.06**	-0.03 ± 0.01^	0.30 ± 0.03**	-0.01 ± 0.01^
Somatization	0.18 ± 0.03**	-0.01 ± 0.01	0.56 ± 0.07**	-0.01 ± 0.01	0.20 ± 0.03**	0.00 ± 0.01

This table presents estimated effects (β ± SE) from linear mixed models examining how baseline amyloid status, neuroticism, and somatization relate to both initial levels and changes over time in depressive and anxiety symptoms. Baseline estimates represent the main effect of each predictor (e.g., Amyloid status), while change-over-time estimates represent the predictor*time interaction (e.g., Amyloid status*Time). All analyses are adjusted for age, sex, and education level. ^p < 0.10, *p < 0.05, **p < 0.01.

GDS, Geriatric Depression Scale; CES-D, Center for Epidemiologic Studies Depression Scale; HADS-A, Hospital Anxiety and Depression Scale – Anxiety.

### 3.2 Associations between personality characteristics and symptoms of depression and anxiety

Higher neuroticism levels were associated with higher baseline GDS, CES-D and HADS-A (β_Neuroticism_ 0.21 ± 0.02; 0.75 ± 0.06; 0.30 ± 0.03; all p<0.001; **Table 2**, **Figure 1B**). Longitudinally, higher neuroticism levels were associated with a slight decrease over time in GDS (β_Time*Neuroticism_ -0.01 ± 0.01, p<0.05), while no associations were observed in CES-D and HADS-A over time. Likewise, higher somatization levels were associated with higher baseline GDS, CES-D and HADS-A (β_Time*Neuroticism_ 0.18 ± 0.03; 0.56 ± 0.07; 0.20 ± 0.03, all p<0.001; **Figure 1C**), but not with change over time in any of the measures.

### 3.3 Effect modification of the association between amyloid status and symptoms of depression and anxiety

**Figure 2:**
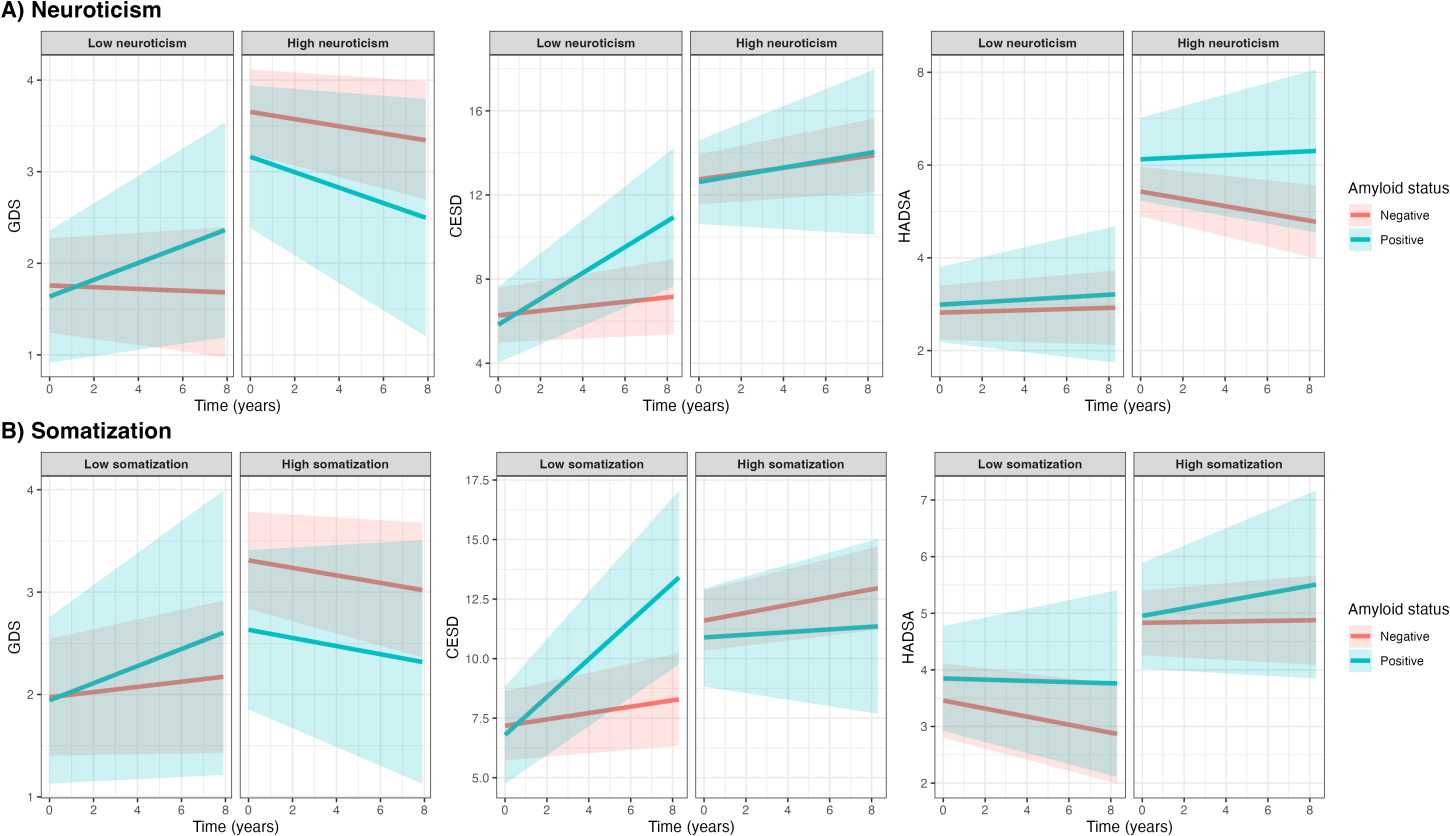
Associations between amyloid status and symptoms of depression and anxiety with personality characteristics as effect modifiers. Figures show symptom trajectories of depression and anxiety over 8 years by amyloid status, based on linear mixed models stratified by low or high neuroticism and somatization (**A, B**, respectively), using median splits (median neuroticism = 5; median somatization = 5). Shaded areas represent 95% confidence intervals. Diverging patterns between high and low groups suggest that neuroticism and somatization may modulate the association between amyloid status and symptoms of depression and anxiety.

Subsequently, we evaluated whether the associations between amyloid status and symptoms of depression or anxiety over time were modified by neuroticism and somatization. Using three-way interaction models, we found that neuroticism modified the association between amyloid status and change in GDS over time (p<0.05; **Table 3**, **Figure 2A**), with a stronger association observed in individuals with low neuroticism levels compared to those with high neuroticism levels. Specifically, amyloid positivity was more strongly associated with an increase in GDS over time compared to amyloid negativity (β_Time*Amyloid status_ 0.10 ± 0.08) in individuals with lower neuroticism, whereas this association was not observed in those with higher neuroticism (β_Time*Amyloid status_ -0.04 ± 0.12). After stratification, we found a similar trend for the association between amyloid status and CES-D over time; the three-way interaction did not reach statistical significance, but its effect size was very similar to the effect size of the interaction term with GDS as outcome (β_Time*Amyloid status*Neuroticism_ -0.04 ± 0.04, p=0.284). Similarly, amyloid positivity was more strongly associated with an increase in CES-D over time compared to amyloid negativity (β_Time*Amyloid status_ 0.50 ± 0.19) in individuals with lower neuroticism, whereas this association was not observed in those with higher neuroticism (β_Time*Amyloid status_ 0.03 ± 0.33).

**Table 3:** Linear mixed model of the association between amyloid status and symptoms of depression and anxiety with personality characteristics as effect modifier.

A: Neuroticism			
Terms	GDS	CES-D	HADS-A
Amyloid status	-0.22 ± 0.43	-0.13 ± 1.05	0.25 ± 0.48
Neuroticism	0.21 ± 0.03**	0.76 ± 0.07**	0.30 ± 0.03**
Time*Amyloid status*Neuroticism	-0.03 ± 0.01*	-0.04 ± 0.04	0.01 ± 0.02
B: Somatization			
Terms	GDS	CES-D	HADS-A
Amyloid status	0.13 ± 0.48	-0.61 ± 1.19	0.27 ± 0.54
Somatization	0.19 ± 0.03**	0.55 ± 0.07**	0.20 ± 0.03**
Time*Amyloid status*Somatization	-0.00 ± 0.02	-0.09 ± 0.05*	0.00 ± 0.02

This table presents estimated effects (β ± SE) from linear mixed models examining the association between amyloid status and trajectories of symptoms of depression and anxiety, with neuroticism and somatization included as effect modifiers. Significant three-way interactions (e.g., Time*Amyloid status*Neuroticism) suggest that the impact of amyloid status on symptom change over time differs depending on levels of these personality characteristics. All analyses were adjusted for age, sex, and education level. ^ p < 0.10, *p < 0.05, **p < 0.01.

Somatization modified the association between amyloid status and change in CES-D over time (p<0.05; **Table 3**, **Figure 2B**), with a stronger association observed in individuals with low somatization levels compared to those with high somatization levels. Amyloid positivity was more strongly associated to an increase in CES-D over time compared to amyloid negativity (β_Time*Amyloid status_ 0.65 ± 0.23) in individuals with low somatization, whereas this association was not observed in those with higher somatization (β_Time*Amyloid status_ -0.12 ± 0.29). The association between amyloid status and HADS-A was not modified by either neuroticism or somatization.

## 4 Discussion

The main finding of this longitudinal study is that there was no simple association between amyloid status and symptoms of depression and anxiety over time in individuals with SCD. Neuroticism and somatization modified the association with symptoms of depression, although not for the association with symptoms of anxiety. Individuals with higher levels of neuroticism and somatization exhibited higher baseline levels of symptoms of depression, regardless of amyloid status, suggesting these symptoms may stem from personality characteristics or other factors. In contrast, in those with lower levels of neuroticism and somatization, amyloid positivity was associated with a steeper increase in symptoms of depression over time. These findings suggest a potential preclinical AD-specific trajectory of symptoms of depression that develops independently of traditional psychiatric risk factors such as neuroticism and somatization.

Research into the influence of personality characteristics on the association between amyloid pathology and symptoms of depression and anxiety in individuals with SCD is limited, with a notable scarcity of longitudinal studies examining the progression of symptoms of depression and anxiety over time. We investigated the association between amyloid pathology and symptoms of depression and anxiety, providing a longitudinal analysis in a relatively large group of individuals with SCD with substantial follow-up. Former findings have been inconsistent, suggesting that other factors also play a role in this association. For this reason, we explored the modulating effects of neuroticism and somatization.

A recent systematic review demonstrated that amyloid pathology was associated with more severe symptoms of depression and anxiety in community dwelling elderly (14). However, studies examining the association between AD biomarkers and symptoms of depression and anxiety in individuals with SCD have been more inconsistent, with some studies reporting associations with more severe symptoms of depression (18, 19), while others did not (17, 20). These inconsistent findings could be partially due to most previous studies being cross-sectional and not accounting for personality characteristics, which we specifically considered in our study. Moreover, the unique nature of the SCD population likely contributes to these inconsistencies. Individuals with SCD, who present with cognitive complaints and worries, likely differ from community-dwelling elderly without SCD. This difference may explain that symptoms of depression and anxiety in SCD might be less directly attributable to AD pathology and more influenced by a broader range of effect-modifying factors, such as personality characteristics, stress responses, maladaptive coping strategies, and pervasive worries. One plausible explanation is that individuals with SCD, particularly those who seek help for cognitive complaints at memory clinics, are likely to have more pronounced concerns, which tend to correlate with higher levels of neuroticism and poorer perceived physical health compared to community-dwelling adults (23, 33). Neuroticism and somatization are well-known personality characteristics and risk factors, which lead to higher degree of depression and anxiety (34, 35). Interestingly, while neuroticism and somatization usually give rise to more psychiatric symptoms and comorbidity, we found a relative stronger increase of depressive symptoms over time in individuals with lower neuroticism and somatization and evidence for amyloid pathology. This suggests state-dependent changes related to a preclinical AD profile in absence of common psychiatric risk factors. While this finding needs further investigation, it is possible that amyloid pathology directly contributes to depressive symptoms as one of the earliest AD-related manifestations. Although the underlying mechanisms are not yet fully understood, amyloid accumulation has been proposed to contribute to affective disturbances through inflammatory pathways, monoaminergic system disruption, or neurocircuitry modifications (21, 22). In contrast, individuals with higher levels of neuroticism and somatization consistently exhibited more severe symptoms of depression and anxiety, regardless of their amyloid status, but these symptoms remained more ‘stable’ over time, which likely illustrates a trait characteristic. This suggests that their symptoms of depression and anxiety may be influenced more by their personality characteristics than by amyloid pathology, underscoring the possibility that higher levels of neuroticism and somatization overshadows or obscures the direct effect of amyloid pathology on the manifestation of depressive symptoms.

Our study is subject to several limitations. First, we excluded all individuals where SCD was primarily explained by psychiatric diseases, including major depression and anxiety disorders. While this allowed us to focus on the association between AD pathology and symptoms of depression and anxiety in a clinically well-defined SCD population, it may limit the generalizability of our findings to the broader older adult population, particularly those with more than subclinical, diagnosed psychiatric disorders. Second, the inclusion of individuals with only subclinical symptoms may have led to an underestimation of the associations between amyloid status and symptoms of depression and anxiety. While we observed moderating effects of neuroticism and somatization, the effect sizes were modest, underscoring the subtle nature of these associations. This may also be partly attributed to sample size limitations or the sensitivity of the measurement instruments, suggesting the need for further investigation in larger and more diverse cohorts. Third, our study did not include a control group of community dwelling elderly without SCD. Therefore, we cannot compare our observed scores on symptoms of depression and anxiety and personality characteristics between individuals with and without SCD. This restricts our ability to fully understand the extent to which these factors are specific to SCD. Nonetheless, the clinical set-up makes our findings a highly relevant for clinical practice.

In conclusion, in individuals with low levels of neuroticism or somatization, amyloid positivity was associated with a greater increase in depressive symptoms over time, suggesting a possible preclinical AD-related depressive profile that exists independently of traditional psychiatric risk factors. In contrast, individuals with high levels of neuroticism or somatization had higher levels of depression regardless of amyloid status, suggesting that their symptoms of depression are more likely to have a different origin, possibly stemming from their personality characteristics or other factors. This underscores the necessity for a personalized approach when assessing and managing symptoms of depression and anxiety in individuals with SCD.

## Data Availability

Data will be shared (anonymized) within the boundaries imposed by the informed consent and data sharing legislation.
